# Reconstruction of a windborne insect invasion using a particle dispersal model, historical wind data, and Bayesian analysis of genetic data

**DOI:** 10.1002/ece3.1206

**Published:** 2014-12-02

**Authors:** Tonya A Lander, Etienne K Klein, Sylvie Oddou-Muratorio, Jean-Noël Candau, Cindy Gidoin, Alain Chalon, Anne Roig, Delphine Fallour, Marie-Anne Auger-Rozenberg, Thomas Boivin

**Affiliations:** 1 INRA, UR629 Ecologie des Forêts MéditerranéennesF-84914, Avignon, France; 2 INRA, UR546 Unité de Biostatistique et Processus SpatiauxF-84914, Avignon, France; 3 National Resources Canada, Canadian Forest Service, Great Lakes Forestry CtrSault Ste Marie, Ontario, P6A 2E5, Canada; 4 INRA, UR633 Unité de Recherche de Zoologie ForestièreF-45075, Orléans, France

**Keywords:** *Cedrus*, HYSPLIT, invasion, long-distance dispersal, *Megastigmus*, microsatellite, wind

## Abstract

Understanding how invasive species establish and spread is vital for developing effective management strategies for invaded areas and identifying new areas where the risk of invasion is highest. We investigated the explanatory power of dispersal histories reconstructed based on local-scale wind data and a regional-scale wind-dispersed particle trajectory model for the invasive seed chalcid wasp *Megastigmus schimitscheki* (Hymenoptera: Torymidae) in France. The explanatory power was tested by: (1) survival analysis of empirical data on *M. schimitscheki* presence, absence and year of arrival at 52 stands of the wasp's obligate hosts, *Cedrus* (true cedar trees); and (2) Approximate Bayesian analysis of *M. schimitscheki* genetic data using a coalescence model. The Bayesian demographic modeling and traditional population genetic analysis suggested that initial invasion across the range was the result of long-distance dispersal from the longest established sites. The survival analyses of the windborne expansion patterns derived from a particle dispersal model indicated that there was an informative correlation between the *M. schimitscheki* presence/absence data from the annual surveys and the scenarios based on regional-scale wind data. These three very different analyses produced highly congruent results supporting our proposal that wind is the most probable vector for passive long-distance dispersal of this invasive seed wasp. This result confirms that long-distance dispersal from introduction areas is a likely driver of secondary expansion of alien invasive species. Based on our results, management programs for this and other windborne invasive species may consider (1) focusing effort at the longest established sites and (2) monitoring outlying populations remains critically important due to their influence on rates of spread. We also suggest that there is a distinct need for new analysis methods that have the capacity to combine empirical spatiotemporal field data, genetic data, and environmental data to investigate dispersal and invasion.

## Introduction

Alien invasive species are a major threat to species survival and can significantly alter entire ecosystems (Gurevitch and Padilla 2004); thus invasion biology is a critical area of research for conservation and landscape management (With 2002). A considerable body of research has focused on how and from where species initially invade (e.g., Wilson et al. 2009b; Guillemaud et al. 2010), and on the ecological and evolutionary processes involved in the establishment success of introduced populations (Lowry et al. 2013). However, investigating the mechanisms and patterns of postestablishment spread (i.e., secondary species expansion) can improve understanding of species’ dispersal abilities, inform management, and help to identify areas where the risk of invasion is highest (Hulme 2006; Dlugosch and Parker 2008).

Secondary expansion of alien invasive species may be investigated directly using empirical data on presence and absence, or indirectly, based on genetic data. Importantly, traditional analysis of either presence/absence or genetic data does not allow statistical testing of which demographic or invasion scenario is the most likely when several are plausible (Guillemaud et al. 2010). Recent advances in population genetics have generated methods to estimate the probability of competing demographic scenarios and reconstruct demographic events such as postestablishment expansion (Estoup et al. 2004). Among these methods is approximate Bayesian computation (ABC), which uses simulations in place of direct evaluation of likelihood functions. The value of ABC methods is that they bypass exact likelihood computation in model fitting by using coalescent-based simulations and summary statistics (Wegmann and Excoffier 2010; Csilléry et al. 2010). In genetic analyses, one of the advantages of ABC methods over more standard methods based on raw measures of genetic distance is that they use controlled simulated data sets to estimate probabilities with credible intervals for each of the demographic scenarios that is being tested, and these probabilities provide a basis a for choosing between scenarios (Cornuet et al. 2008; Guillemaud et al. 2010). ABC has been particularly useful in comparing the probability of competing demographic scenarios and estimating population parameters given complex demographic histories. It has recently been used in estimating the rate of spread of pathogens (Shriner et al. 2006) and in reconstructing routes of introduction for invasive species (e.g., Guillemaud et al. 2010; Lombaert et al. 2010).

In this study, we used ABC to jointly analyze presence/absence, genetic and wind data to understand the invasion and expansion of a highly invasive insect from the seed-specialized microhymenoptera (Torymidae), a group which presents a major ecological and economic threat in Europe (Roques 2010; Auger-Rozenberg and Roques 2012). *Megastigmus schimitscheki* is a predator of true cedar seeds, *Cedrus atlantica* (Pinaceae) in France and *C. brevifolia* and *C. libani* in its native range of Asia Minor. Introduced populations of the wasp can have a direct impact on the reproductive success of their obligatory host by reducing viable seed production by up to 87% (Fabre et al. 2004; Boivin et al. 2008).

Annual surveys of cone and seed pests in French *C. atlantica* stands have been conducted since 1978, and in 1994, it revealed the first specimens of *M. schimitscheki*. The wasp was introduced to France only once, between 1990 and 1993, from Cyprus to the region of Mont Ventoux through clandestine *C. brevifolia* seed trade for ornamental purposes. French populations were founded from an extremely restricted number of individuals that realized a host shift from *C. brevifolia* to *C. atlantica* (Auger-Rozenberg et al. 2012). Importantly, its new host plant *C. atlantica* is also not native in France, but it has been planted for timber and therefore shows a patchy distribution with inter-patch distances frequently greater than 10 km, which is further than the documented active flight range in the *Megastigmus* genus (Jarry et al. 1997). However, by 2006, *M. schimitscheki* had been identified at sites 110 km away from Mont Ventoux (Suez et al. 2013), suggesting vector-assisted dispersal.

**Figure 1:**
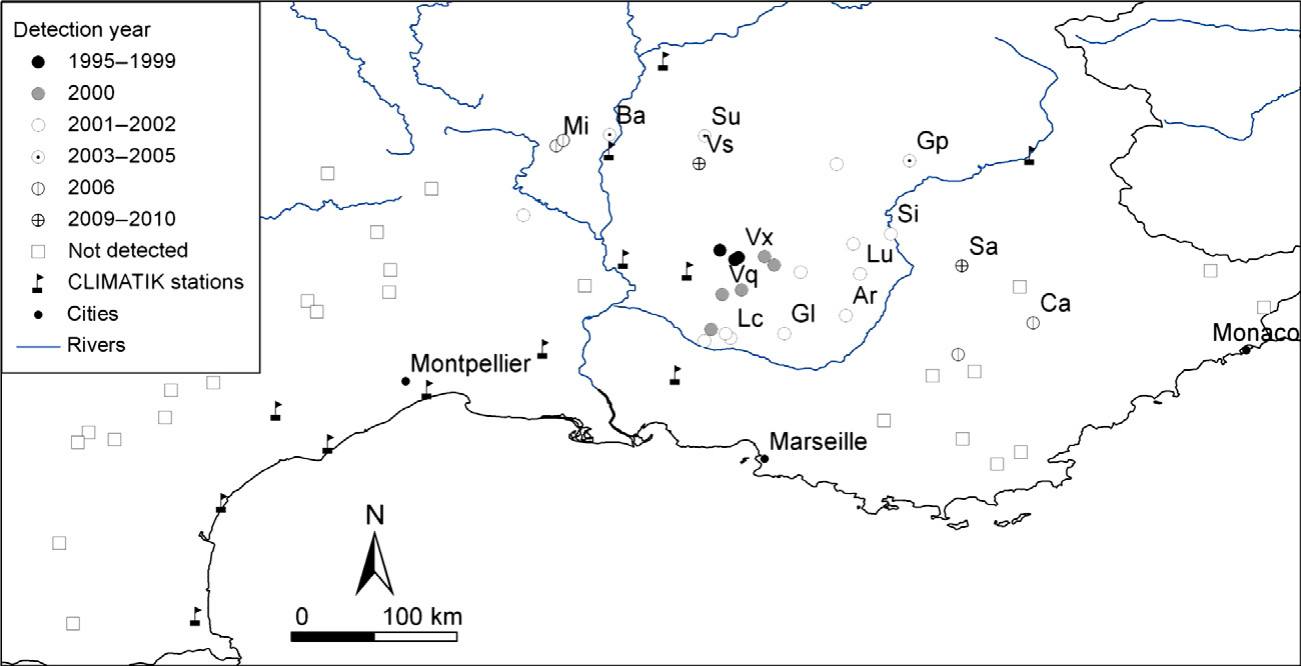
The survey sites showing the year of arrival and the CLIMATIK weather stations used in the DIYABC analysis. All sites included in the annual survey (Table S1) are included on this map, but only those sites where *Megastigmus schimitscheki* specimens were collected for the genetic analysis have name codes associated with them. These study sites are shown as: Ardene (Ar), Barrs (Ba), Castellane (Ca), Gap (Gp), Grand Luberon (Gl), Luberon Crete (Lc), Lure (Lu), Mirabel (Mi), Saou (Su), Saumon (Sa), Sisteron (Si), Venasque (Vq),Ventoux (Vx), Vesc (Vs). Survey sites where *M. schimitscheki* has not been detected are shown as “not detected”.

The spread of *M. schimitscheki* across southeastern France has not been equal in all directions away from Mont Ventoux; instead, the majority of the infested stands are located to the east or south-east of Mont Ventoux (Fig. 1). The Rhone valley has intermittent strong winds, known as the Mistral, which travel toward the southeast and are known to be important in the movement of aerosol particles (Salameh et al. 2007). Prevailing winds have been known to contribute to colonization by other species (e.g., Venette and Ragsdale 2004; Whitmire and Tobin 2006; Anderson et al. 2010), and the *Megastigmus* genus is prone to windborne dispersal (Jarry et al. 1997); we therefore investigated the possible influence of wind on patterns of *M. schimitscheki* colonization in southern France.

The extensive annual survey data for *M. schimitscheki* across southeastern France provides the opportunity to address three questions:

1. Does *M. schimitscheki* spread through short-distance ‘stepping-stone’ dispersal (e.g., Shigesada and Kawasaki 1997; Ciosi et al. 2010) or long-distance dispersal (e.g., Hastings et al. 2005; Nathan et al. 2008) from the area of introduction?
2. Can we detect the influence of either local-scale and near ground-level wind data or regional-scale and higher altitude wind in the observed patterns of *M. schimitscheki* colonization?
3. Did patterns of secondary dispersal leave a signature in the spatial genetic structure of the *M. schimitscheki* populations?

For question 1, we tested several hypothetical demographic scenarios based on short- versus long-distance dispersal using two independent and complementary approaches: (1) analysis in the ABC-based program DIYABC (Cornuet et al. 2008) which compares the probability of competing demographic scenarios based on genetic data, and (2) survival analysis based on *M. schimitscheki* presence/absence data at the study sites over the sample period. For question 2, we used the DIYABC program to compare hypothetical demographic scenarios based on local or regional wind data with a scenario that was not based on wind data. For question 3, we combined several classical analyses of population structure, some of which included spatial position of individuals or populations in the analysis.

We had the opportunity to conduct this study in a system where the area of *M. schimitscheki* introduction is known and the year of introduction may be closely estimated. In addition, we have presence/absence data for the study species at 52 sites across southeastern France for the 16 years since establishment, and extensive genetic data. This study allowed us to estimate the influence of local-scale and regional-scale wind movement on organism dispersal, evaluate the probability that both new and long-established sites serve as source populations for invasive species spread and assess the efficacy of current seed pest survey methods. Based on our results, we provide suggestions for invasion control and monitoring.

## Materials and Methods

### Study species

In France, the introduced species *M. schimitscheki*, from Asia Minor, co-exists with the conspecific *M. pinsapinis*, which was introduced from North Africa at the end of the nineteenth century (Fabre et al. 2004). Both species are obligate predators of true cedar seeds (*Cedrus* spp.). The wasps lay their eggs inside cedar ovules by inserting their ovipositor through young cone scales, and the larval instars develop by consuming the seed (Rouault et al. 2004). In France, adult *M. schimitscheki* emerge over a 15-day period in mid-May and *M. pinsapinis* emerge 9–18 days later (Boivin et al. 2008). The individual flight period for both species is assumed to be 10 days as for *M. spermotrophus* (Jarry et al. 1997). The successful invasion of *M. schimitscheki* is thought to have been supported by advantageous life-history traits, especially phenology, compared with *M. pinsapinis* (Boivin et al. 2008), and annual variation in the supply of cedar seeds (cedar masting cycles), because during years with high-seed abundance competition between the two species may be relaxed (Auger-Rozenberg et al. 2012). This study did not involve any endangered or protected species.

In southeastern France, cedar stands are patchily distributed in the landscape with interpatch distances frequently greater than 10 km. Because the *Megastigmus* genus has not been shown to actively travel over distances as great as 10 km (Jarry et al. 1997), but *M. schimitscheki* appears to have spread approximately 110 km away from Mont Ventoux in the eleven years between introduction and detection at the most distant site, Castellane (Fig. 1), wasp dispersal between cedar stands is likely to be passive. Within cedar stands, *Megastigmus* species actively fly between trees to forage for resources (Jarry et al. 1997), and continue foraging even when there is strong wind (T. Boivin, unpubl. data). Models of dispersal for this genus have suggested that windborne passive dispersal may occur when individuals fly to higher altitudes where there are stronger winds and air turbulence (Jarry et al. 1997; Roques et al. 2008), but whether adults can be passively dispersed by wind at lower altitudes is still unknown.

### Field surveys

Surveys for *M. schimitscheki* were conducted between 1995 and 2010 at 52 field sites across the species’ known range in France (Fabre et al. 2004) (Fig. 1, Table S1). At each study site, consisting of a pure cedar or mixed cedar-fir (*Abies* spp., Pinaceae) stand, three mature cones were randomly collected at 2 m above the ground on 10 randomly selected cedar trees before seasonal cone disarticulation to estimate seed infestation rate by *Megastigmus* spp. In the laboratory, cones were disarticulated and seeds colonized by either of the two *Megastigmus* species were identified by Faxitron numerical X-ray radiography (Faxitron Bioptics, Tucson, AZ; 15–20 kV, 0.3–3 mA), grouped according to year and site of collection, and stored under natural conditions. Adults emerging in the spring were determined to species, sexed and stored in 100% ethanol for genetic analysis (see Boivin et al. 2008).

### Genetic data

A total of 661 individuals from 14 study sites (Table 1) were genotyped at nine microsatellite loci. For DNA extraction, each insect was placed in a 200-*μ*L tube with 10% chelex resin 100 and 6 *μ*L 10 mg/mL proteanase K (Walsh et al. 1991). The insects were macerated in the tubes using a QIAGEN TissueLyser (QIAGEN Ltd, Crawley, UK; two runs, 10 sec, 20 Hz). The tubes were incubated at 56°C for 2.5 h. After incubation, the tubes were heated twice to 100°C for 15 min to stop the enzymatic reaction. The DNA extract was stored at −20°C. Before the PCR, the tubes were heated to 100°C for 1 hr and then centrifuged at 4000 rpm to pellet the chelex. About 10 *μ*L of the supernatant containing the DNA was diluted in 30 *μ*L H_2_0.

**Table 1:** The study sites used in the genetic analyses. Number of individuals (*N*), Mean number of alleles at 9 SSR loci (Na), Allelic richness (minimum sample size of 9, *A*_R_), gene diversity (*H*_E_), and fixation index (*F*_IS_). None of the *F*_IS_-values were significant.

Study site	Latitude	Longitude	Elevation	*N*	Na	*A*_R_	*H*_E_	*F*_IS_
Ardene	43.89	5.73	495	30	3.3	2.738	0.442	−0.012
Castellane	43.86	6.52	1005	32	3.5	3.076	0.552	0.061
F.D. Lure	44.07	5.79	960	30	3.1	2.739	0.497	0.008
F.D. Venasque	43.98	5.21	∼320	30	3.3	2.940	0.531	−0.024
Foret de Barres	44.66	4.73	470	9	2.3	2.500	0.417	−0.173
Gap	44.55	6.00	990	30	3.0	2.759	0.456	0.020
Grand Luberon	43.81	5.47	1100	30	2.9	2.813	0.513	−0.031
Luberon crete	43.80	5.24	670	278	5.6	3.097	0.491	0.083
Mirabel	44.61	4.51	590	46	2.7	2.159	0.324	0.413
Mont Ventoux	44.14	5.39	1085	30	3.6	3.238	0.558	0.072
Saou	44.65	5.13	475	30	3.0	3.159	0.539	0.184
Saumon	44.10	6.17	∼600	27	2.5	2.694	0.482	0.060
Sisteron	44.24	5.92	500	29	3.1	3.116	0.522	0.246
Vesc	44.53	5.11	∼655	30	3.1	2.846	0.482	−0.099
Mean all sites					3.2	3.557	0.486	

Each PCR contained 2 *μ*L diluted DNA, 1 *μ*L Q-solution(5×), 1 *μ*L H_2_O, 5 *μ*L QIAGEN Multiplex PCR Master mix 2× (6 mM MgCl_2_, HotStar Taq, dNTP mix), and 1 *μ*L of 2 *μ*M primer mix. PCRs were conducted as two multiplex reactions using genus-specific microsatellite primers (Table S2, Carcreff et al. 1998; Boivin et al. 2003). The PCR program was 95°C 15 min, 30× (94°C 30 sec, 57°C 90 sec, 72°C 90 sec), final extension 72°C 10 min in an Eppendorf MasterCycler thermocycler (Eppendorf UK Ltd, Stevenage, UK). Alleles were sized on a MegaBACE 1000 sequencer using MegaBACE ET400-R Size Standard (GE Healthcare-Amersham Bioscience, Little Chalfont, UK). The microsatellite profiles were analyzed using a MegaBACE Genetic Profiler v.2.2 (GE Healthcare-Amersham Bioscience, Little Chalfont, UK). The probability that two individuals that were not genetically identical would show the same genotype (the probability of identity) using the nine loci employed for this study was 4.7 × 10^−7^ (GenAlEx 6.4, Peakall and Smouse 2006).

### Genetic diversity and differentiation

The following standard population genetic statistics were calculated within each study site in FSTAT 2.9.3.2 (Goudet 2001): mean number of alleles (Na), gene diversity (*H*_E_), allelic richness (*A*_R_), and inbreeding coefficient (*F*_IS_). The significance of *F*_IS_-values was assessed at the 5% confidence level using strict Bonferroni corrections. Allelic richness was estimated using rarefaction with a sample size of nine individuals (minimum sample size per plot). The components of variance in allelic frequencies among study sites and among individuals were estimated using a hierarchical AMOVA in ARLEQUIN v.2 (Excoffier et al. 2005). In the case of microsatellites following the stepwise mutation model, Slatkin (1995) recommends the *R*_ST_ statistic which measures variation in allelic frequencies and genetic differentiation with alleles ordered according to their size rather than with unordered alleles (identity in state). Accordingly, we calculated both *F*_ST_ and *R*_ST_ in ARLEQUIN as estimators of genetic differentiation between groups and populations. All loci were tested for significant deviation from Hardy–Weinberg equilibrium in ARLEQUIN. Null allele (NA) frequencies were estimated for each locus in each population according to the Expectation Maximization algorithm in FreeNA (Chapuis and Estoup 2007).

We tested whether geographic distances between populations explained the patterns of genetic structure by computing *F*_ST_ / (1 − *F*_ST_) and *R*_ST_ / (1 − *R*_ST_) between all pairs of sampling plots, computing the regression slope of those values against the logarithm of pairwise geographic distance (b_log_*F*_ST_ or b_log_*R*_ST_), and comparing these slopes to their frequency distribution under the null hypothesis of spatial genetic randomness obtained by 5000 permutations of individuals among populations (Hardy and Vekemans 2002). The geographic distance values were Euclidian distances between plots calculated in ArcView 3.1 (ESRI, Redlands, CA). To determine whether stepwise-like mutations contributed to genetic differentiation (whether *R*_ST_ = *F*_ST_), we used the test in SpaGeDi (Hardy and Vekemans 2002) proposed by Hardy et al. (2003) which is based on a randomization of allele sizes.

A further test of genetic structure was conducted using Bayesian clustering of the genetic data in STRUCTURE v.2.3.3 (Pritchard et al. 2000). We ran STRUCTURE with K varying between 1 and 10, with 10 runs for each K value. We used 10,000 burn-in runs and 100,000 MCMC repetitions after burn-in. Four sets of parameters were applied (allele frequencies were correlated among populations in all four): (1) user-defined sampling location (population identity) was used to assist the clustering, and no admixture was included in the model of population structure; (2) no population identity and no admixture (3) population identity and admixture; (4) no population identity and admixture. The optimal number of clusters (K) represented by the data was determined with the method described by Evanno et al. (2005).

### Wind data

We used wind data from two sources, local-scale wind data from the CLIMATIK database (INRA AGROCLIM 2011) and regional-scale wind data from the United States National Oceanographic and Atmospheric Association's (NOAA) HYSPLIT model (Draxler and Rolph 2003).

#### Local-scale wind data

Wind data were retrieved from the CLIMATIK database for 12 weather stations in southeastern France for each day in May for the years 1994–2010 (Fig. 1, Table S3). The data were for May because adult *M. schimitscheki* live and disperse exclusively in May in southeastern France (Boivin et al. 2008). The wind data consisted of the quantity of wind (the daily distance in kilometers travelled by wind from a given direction) at a maximum of 10 meters above the ground traveling in each of the eight cardinal and subcardinal directions during May of each year from 1994 to 2010. We refer to the CLIMATIK data as “local-scale” because the wind data retrieved from each weather station provided information on local variation in wind direction whereas in the regional-scale wind model local variation in wind activity is not included.

#### Regional-scale wind data

The NOAA HYSPLIT model uses historic wind data to estimate the flight trajectories of particles hypothetically released from a given location on a given historical date and allowed to travel for a specified period of time anywhere in the world (Draxler and Rolph 2003). Within the HYSPLIT model, we used the National Centers for Environmental Prediction and National Center for Atmospheric Research dataset (NCEP/NCAR Reanalysis: 1948–2010) and ensemble trajectories. Particle release height was 50 m to simulate high-altitude insect dispersal, although the release height does not restrict the altitude to which particles may travel, either up or down, along their dispersal trajectories. Particles were allowed to travel for 15 h from a release time of 6:00 (approximate sunrise) until 21:00 (approximate sunset) to mimic the diurnal flying activity of adult wasps. Each trajectory was re-estimated every hour (Figure S1). Trajectories were modeled for departure from each of the study sites for each day in May every year beginning with the year colonization was detected at each site (total of 6014 days modeled at 23 sites; Table S4). The horizontal resolution of the NCEP/NCAR Reanalysis data is T62 (approximately 210 km) across the Earth's surface. Because the study area is approximately 250 km by 400 km, this means that the same “cell” of model data was used to estimate trajectories from most or all of the study sites, thus any local variation in wind direction or speed within the study area is not included in the NCEP/NCAR database and would not have been included in the modeled data.

It should be noted that we did not include information on temperature or precipitation at the study sites in our analyses. Although these factors can affect insect activity, our CLIMATIK database (1994–2010) indicated that diurnal temperatures in southern France in May always remain above the thermal threshold of adult flight activity (ca. 16°C) and that the annual number of days with precipitation varied from zero to three. We therefore assume that the influence of these factors is negligible in this study.

### Survival analyses of presence-absence data

We used survival analyses to test whether long-distance dispersal from the point of introduction or short-distance “stepping-stone” dispersal better explained observed patterns of colonization. Survival analysis is a statistical method typically used to analyze the probability of death in biological organisms exposed to a treatment (Klein and Moeschberger 2003). In this study, the event corresponding to “death” was the colonization of a site by *M. schimitscheki* as observed in the annual surveys. The cumulative exposure to “treatment” was the annual arrival of *M. schimitscheki* individuals at the site, which was modeled as the yearly HYSPLIT particle point count in the grid cell of each study site (see below). There was no equivalent quantitative value that could be derived from the CLIMATIK data so we did not conduct a survival analysis of the local-scale wind data.

**Figure 2:**
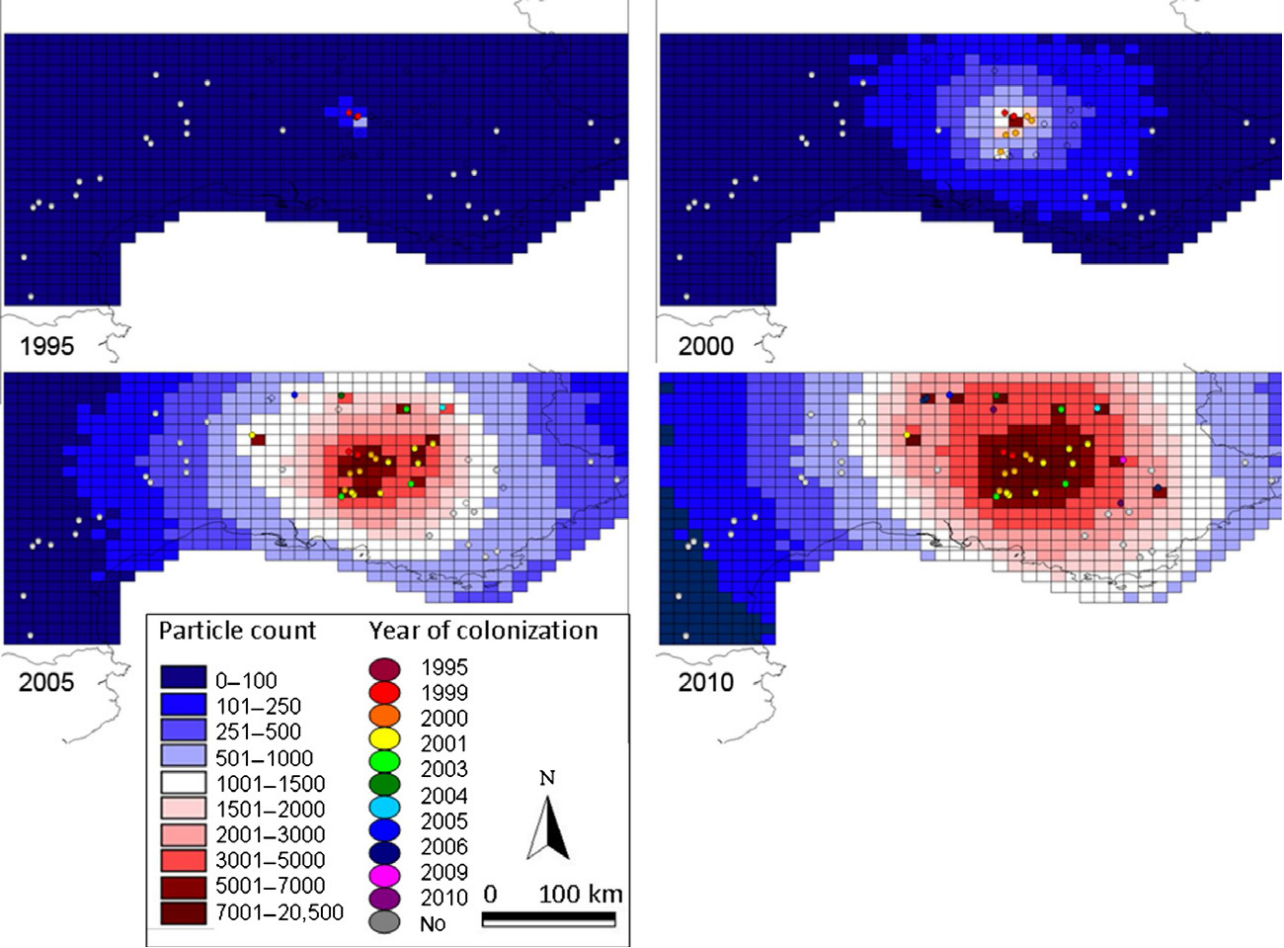
Probability of colonization by *Megastigmus schimitscheki* estimated based on the number of HYSPLIT wind-dispersed particle trajectory point estimates in each 10 km × 10 km cell across southeastern France. The NOAA- HYSPLIT wind-dispersed particle trajectory model was used to model the travel paths of particles (representing passively dispersed wasps) released from each study site every day in May each year following colonization of each site (for model results for dispersal from a single study site on a single day see Figure S1). Three subsets of this particle trajectory data were overlaid on a grid of 10 km × 10 km cells covering the study area to create the scenarios for the DIYABC and Survival analyses: (1) *Long-distance dispersal from Mont Ventoux*: only those trajectories originating at Mont Ventoux, the apparent site of *M. schimitscheki* introduction to France, (2) *Short-distance dispersal from all sites:* trajectories originating from any colonized study site starting from the year the site was identified as colonized during the annual surveys, and (3) *Short-distance dispersal from all sites plus 4-year delay:* trajectories originating from any colonized study site starting 4 years after the site was identified as colonized during the annual surveys. For each of these scenarios, the number of particle trajectories which passed over each grid cell was counted and summed from 1 year to the next to create an estimate of the probability of colonization at each study site in each year. This figure shows the sum of particle trajectories passing over each grid cell using the *Short-distance dispersal from all sites* scenario in years 1995, 2000, 2005, and 2010. The estimated year of colonization for each study site is also shown.

We overlaid the regional-scale HYSPLIT particle trajectory data onto a grid of 10 km × 10 km cells covering the study area in ArcView 3.1 (Fig. 2). Of 10 km^2^ grid cells were used because the maximum distance between HYSPLIT one-hour point estimates was 10 km, thus a grid cell would not be missed because it fell between point estimates along a trajectory. To estimate the probability of colonization in each grid cell in each year, the number of particle point estimates that fell within each grid cell was counted and summed from 1 year to the next using three different scenarios:

1. *Long-distance dispersal from Mont Ventoux*: Only trajectories originating from Mont Ventoux, the initial introduction site and the long-established population, were used to generate point counts in the grid cells. In this scenario, colonization of the study sites was modeled as occurring only through long-distance dispersal from Mont Ventoux. This meant that in all years colonization probability increased with proximity to Mont Ventoux.
2. *Short-distance dispersal from all sites*: For this scenario, each study sites was considered “colonized” starting in the year the site was actually identified as colonized during the annual surveys, thus the number of sites considered colonized increased from year to year in this scenario. Trajectories originating from all colonized study sites were used to generate point counts in the grid cells in each year of the scenario. The probability of colonization of each study site increased as neighboring sites were colonized because sites that were colonized became sources of emigrants; therefore, several years after the initial introduction, the probability of colonization was no longer directly linked to proximity to Mont Ventoux. This scenario therefore models colonization as occurring through short-distance dispersal in a stepping-stone pattern between study sites.
3. *Short-distance dispersal from all sites plus 4-year delay*: Trajectories originating from any colonized study site, starting 4 years after the site was identified as colonized during the annual surveys, were used to generate point counts in the grid cells. This third scenario models colonization as occurring through short-distance stepping-stone dispersal, as in *Scenario 2: Short-distance dispersal from all sites*, and in addition, it allows for delay in the time it takes for a population to grow large enough within a site to become an important source of emigrants to other sites. Four years was used as the delay time because Mont Ventoux appears to have become a source of emigrants in 1995, approximately 4 years after the initial population was introduced (Auger-Rozenberg et al. 2012).

The explanatory power of the three scenarios above was tested using time-dependent survival analysis. We tested the effect of yearly particle point count in the grid cell of each study site on the rate at which establishment of migrants occurred at that study site. The only explanatory variables for the survival analysis were based on counts of the regional-scale HYSPLIT particle trajectories. Our data were interval-censored, meaning that we did not observe the exact time at which first colonization occurred but could only observe that it occurred within an interval of years. We used time-dependent covariates (Klein and Moeschberger 2003; Soubeyrand et al. 2007; see Table S1 for a precise description of the tested data). We assumed that successful establishment of *M. schimitscheki* migrants in a site occurred following a simple Poisson time-process with time-varying intensity, and we implemented a maximum likelihood estimation in MATHEMATICA 8.1 (Wolfram Research, Long Hanborough, UK)) (see Table S5 for details).

Rather than estimating a dispersal kernel for the wasps, in which the probability of descending from altitude and landing on a host tree would have been higher closer to the point of origin, we assumed that there was an equal probability that a wasp would descend from altitude at each hourly point estimate along each of the particle trajectories. This is because even if the wasps are passively dispersed by the wind, there is evidence that insects actively descend from or remain in the air column in response to the presence or absence of appropriate hosts (Nason et al. 1996), although the mechanisms for passive wind dispersal combined with active location of specific hosts are not well understood (Reynolds and Reynolds 2009). Of course it should be noted that there is also evidence that some insects are simply passively dispersed and may be carried far beyond the limits of appropriate landing sites (Elton 1925).

### ABC analyses of genetic data

We used an ABC approach implemented in the program DIYABC 1.0 (Cornuet et al. 2008) to test whether *M. schimitscheki* colonized through short or long-distance dispersal and whether we could detect the influence of either local-scale and near ground-level wind data or regional-scale and higher altitude wind in the observed patterns of *M. schimitscheki* colonization. DIYABC uses approximate Bayesian computational methods to analyze microsatellite genetic data, compute posterior probability associated to several proposed demographic scenarios, and estimate demographic parameters.

#### Definition of scenarios

**Figure 3:**
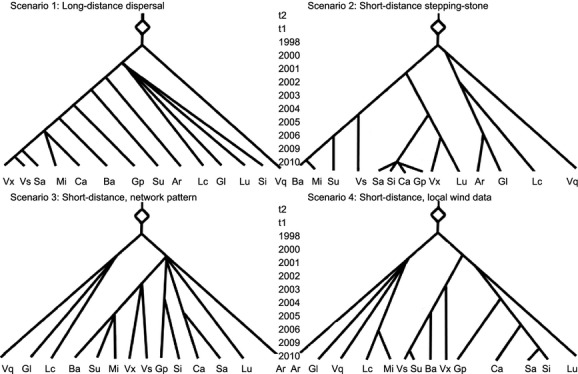
The four scenarios used in the DIYABC analysis. The study sites are shown as: Ardene (Ar), Barres (Ba), Castellane (Ca), Gap (Gp), Grand Luberon (Gl), Luberon Crete (Lc), Lure (Lu), Mirabel (Mi), Saou (Su), Saumon (Sa), Sisteron (Si), Venasque (Vq),Ventoux (Vx), Vesc (Vs).

We tested four alternative scenarios of *M. schimitscheki* dispersal away from Mont Ventoux: (1) *M. schimitscheki* colonized through long-distance dispersal, independently of wind patterns; (2) *M. schimitscheki* colonized through short-distance dispersal, based on a stepping-stone model calibrated by regional wind data; (3) regional-scale and higher altitude wind data explain observed patterns of colonization; (4) local-scale and near ground-level wind data explain observed patterns of colonization. These four demographic scenarios are presented as coalescent “gene genealogies,” similar to phylogenetic trees but representing the process whereby individuals from an established population emigrate and establish a new population, (Fig. 3). All four scenarios are characterized by (1) “time steps” (measured in generations, which are single years) which are points in time when immigrants from one population could establish a new population, (2) the number of populations present at each time step and (3) the effective population size. One should note that we did not infer though the ABC procedure the sets of parameters corresponding to the years of colonization of each site, rather we fixed these parameters a set of values varying among the different scenarios. At generation 0, the present time, all of the scenarios had fourteen populations corresponding to the fourteen study sites from which we had genetic data. The identification of these study sites as distinct populations was based on spatial separation and significant pairwise *F*_ST_ (see Results). No single nucleotide in-del mutations were allowed in the simulations, the Generalized Stepwise Model of SSR mutation was assumed, and the mean SSR mutation rate ranged between 1 × 10^−4^ and 1 × 10^−5^. According to Chapuis et al. (2011), these SSR mutation rates allow a range of 1 × 10^−3^ to 1 × 10^−6^ for single-locus mutation rates which encompasses current estimates for insects.

The four demographic scenarios for DIYABC analysis were as follows, and they are explained in more detail immediately after they are introduced (Fig. 3):

1. *DIYABC Scenario 1, Long-distance dispersal*: All of the colonized sites received their immigrant population through long-distance dispersal directly from Mont Ventoux, the introduction site of the wasp, and no other site acted as a source population. The year of colonization was assumed to be the year of detection in the annual surveys. This scenario did not consider the influence of either local or regional wind patterns.
2. *DIYABC Scenario 2*, *Short-distance stepping-stone dispersal*, *regional wind data*: The year of colonization for each of the sites was estimated based on the regional wind data, which was used to create a stepping-stone dispersal scenario. In this scenario, Mont Ventoux was not assumed to be the only source of emigrants, but only wind data from Mont Ventoux was used. In this way, the yearly rate of spread of *M. schimitscheki* out from Mont Ventoux is slowed compared with the method used in *DIYABC scenario 3*, and annual colonization moves outwards from Mont Ventoux in a strict stepping-stone pattern, but any colonized site may be a source of emigrants. The source of colonization for a given site was the nearest neighbor site with an already established population.
3. *DIYABC Scenario 3*, *Short-distance network pattern dispersal*, *regional wind data*: The year of colonization for each of the sites was estimated based on the regional wind data. In this scenario, as additional sites become sources of emigrants the pattern of colonization across the study landscape forms a network, in contrast to the strict stepping-stone pattern in *DIYABC scenario 2*. The source of colonization for a given site was the nearest neighbor site with an already established population.
4. *DIYABC Scenario 4, Short-distance dispersal, local wind data*: The source of colonization for each site was based on the local wind data, but the year of colonization was the year when colonization was detected in the annual surveys. The source of colonization for a given site was the nearest neighbor site with an already established population.

For DIYABC scenarios 2 and 3 which use the regional wind data, we assumed that the annual seed pest surveys might not have detected colonization at the study sites in the first year that there was *M. schimitscheki* present. Therefore, the year of colonization for each site was estimated as follows: on the grid of 10 km^2^ cells covering the map of the study area (Fig. 2), we calculated the average regional-wind trajectory particle point count in all cells with study sites in the year before *M. schimitscheki* was detected in the annual survey. This was performed using the *Long-distance dispersal from Mont Ventoux* and the *Short-distance dispersal from all sites* scenarios described in the “Survival analyses of the presence/absence data” section above for DIYABC scenarios 2 and 3, respectively. The particle point count was rounded to 200 for the *Long-distance dispersal from Mont Ventoux* scenario and 800 for the *Short-distance dispersal from all sites* scenario. These point count values were used as thresholds so that the year a cell containing a study site reached the *Long-distance dispersal from Mont Ventoux* or *Short-distance dispersal from all sites* threshold value was used as the estimated year of colonization for DIYABC scenarios 2 and 3 respectively. Because the regional-scale wind model data used in DIYABC scenarios 2 and 3 indicated that in all years at all sites, there was some probability that wind travelled in each of the eight cardinal and subcardinal directions at least 1 day in May, we could not predict the source population based on wind direction. Therefore, the source population for each site was the nearest neighbor site with an already established *M. schimitscheki* population because short-distance dispersal is generally more common than long-distance dispersal (Nathan et al. 2008) (Table S6).

For DIYABC scenario 4 which used local-scale wind data, the direction from each study site to every other study site was categorized as one of the cardinal or subcardinal directions (e.g., North is identified with all angles from 337.5 to 22.5 degrees); the wind data from the nearest CLIMATIK weather station were used for each source site (Fig. 1), and those data were categorized as present or absent in each of the cardinal or subcardinal directions; and finally the year when *M. schimitscheki* was detected at a given site in the annual survey was assumed to be the year after it arrived at that site. The probability that immigrant *M. schimitscheki* colonized a new site from any one of the already colonized sites was based on the presence or absence of wind in the direction from the potential source site toward the uncolonized site in May of the year prior to detection at the uncolonized site. If multiple source sites appeared to be possible sources of colonization, the source site nearest to the uncolonized site was selected.

#### Prior distributions and summary statistics

Defining the prior distribution of parameters is a key step in ABC analysis. Distributions should include all of the values which are considered possible and prior information can be based on previous knowledge of some parameters (Bertorelle et al. 2010). We know from routine field sampling that *M. schimitscheki* population size increases with time as 12 years after introduction local abundance of the wasp was found to be tenfold higher in the introduction area than in newly colonized areas (Boivin et al. 2008; Suez et al. 2013). The prior distributions for effective population size were thus set at (10–10,000) for wasp populations detected in 1995–2001 (i.e., more than four generations before sampling), at (10–5000) for those detected in 2002–2006 (i.e., two to four generations before sampling), and at (10–1000) for those detected in 2009–2010 (i.e., one generation before sampling). Because of the lack of precise knowledge of population size, we chose a uniform distribution for effective population size in these three population groups. Extensive field monitoring of cedar seed infestation since 1978 has allowed us to establish that the French *M. schimitscheki* population was founded between 1990 and 1993, which led us to use a uniform distribution with a narrow interval (12–15 generations) for foundation time of the Mont Ventoux population (t1) in the models. The other dates of divergence were fixed and not estimated. We modeled the Mont Ventoux population as the result of recent admixture between two populations to take into account the observed genetic diversity in the initial population on Mont Ventoux and allow for the possibility that the population was not in Hardy–Weinberg equilibrium. A list of both demographic and time parameters and prior distributions used to model scenarios is summarized in Table S7.

In the DIYABC analysis, the statistics summarizing the *M. schimitscheki* microsatellite data were Nei's allelic diversity and mean Garza-Williamson's M (Garza and Williamson 2001). For each of the pairs of populations, we used the Classification Index (Rannala and Mountain 1997). The selection of which and how many summary statistics should be used in ABC is still a matter of debate (Lombaert et al. 2011). Although recent improvements in ABC analysis include alternative dimension reduction techniques to address this problem (Wegmann et al. 2009; Blum and Francois 2010), these techniques are mainly devoted to the estimation of posterior distributions of demographic parameters under a given scenario and not to discrimination between competing scenarios (Lombaert et al. 2011), whereas discrimination between competing scenarios was the goal of the present study.

#### Simulations and posterior probability estimation

One million data sets were simulated for each scenario (as recommended in Cornuet et al. 2008, 2010). The posterior probabilities of the four DIYABC scenarios were estimated using polychotomous logistic regression of each scenario probability on the deviations between simulated and observed summary statistics, as implemented in DIYABC (Cornuet et al. 2008). The selected scenario was the one with the highest probability value with a nonoverlapping 95% confidence interval. Confidence in scenario choice was evaluated by computing type I and type II errors in the selection of scenarios. Type I errors were computed as the proportion of times that a given scenario did not have the highest posterior probability when it was the true scenario, and type II errors were computed as the proportion of times that a scenario had the highest posterior probability when it was not the true scenario (Cornuet et al. 2008). Within DIYABC, we also performed a principal component analysis (PCA) in the space of the summary statistics in which the observations were 5000 simulated data sets and the variables were the summary statistics. Goodness-of-fit of the selected DIYABC scenario was assessed based on the location of the PCA points simulated from the posterior predictive distribution compared to that of the point corresponding to the observed genetic data (Cornuet et al. 2008). We used different summary statistics for model checking than for computations of parameter posterior distributions: the mean number of alleles for each population, the mean allele size variance, the shared allele distance and the distance (*δμ*)² between all pairs of populations. This allowed us to avoid an overestimation of scenario fit to our data (Cornuet et al. 2010).

## Results

### Genetic diversity and differentiation

Levels of diversity as measured by Nei's heterozygosity (He) or allelic richness (A) were similar in the fourteen populations, with overall means of He = 0.486 and A = 3.557 (Table 1). The spatial hierarchical AMOVA analysis using either *F*_ST_ or *R*_ST_ showed that most of the genetic variation was among individuals within populations, but there was also significant genetic differentiation between populations (*F*_ST_ = 0.1721; Table 2, Table S8). *F*_IS_ and *R*_IS_ were not significant using a 95% confidence interval, but *F*_ST_ and *R*_ST_ were significant (Table 1, Table S8). There was also significant pairwise *F*_ST_ between most of the populations (Table 2). None of the loci showed significant departure from Hardy–Weinberg equilibrium.

**Table 2:** Pairwise *F*_ST_ values. Those values not significant after Bonferroni correction are italic and bold.

	Vesc	Saumon	Mirabel	Castellane	Foret de Barres	Gap	Saou	Ardene	Luberon crete	Grand Luberon	F.D. Lure	Sisteron	F.D. Venasque
Mont Ventoux	0.1938	0.2221	0.2893	0.1552	0.0791	0.2475	0.0220	0.2556	0.1854	0.0365	0.1989	0.0397	0.1765
Vesc	–	0.1283	0.2856	0.1437	0.2462	0.1622	0.1966	0.1898	0.1711	0.2145	0.1868	0.1950	0.1684
Saumon		–	0.3154	0.1688	0.2478	0.2499	0.1896	0.2164	0.2531	0.2352	0.1950	0.2294	0.2043
Mirabel			–	0.2245	0.1981	0.3453	0.2270	0.2226	0.1684	0.3192	0.1569	0.2776	0.2154
Castellane				–	0.1986	0.1245	0.1539	0.1128	0.1141	0.1434	0.0754	0.1608	0.0794
Foret de Barres					–	0.3458	***0.0325***	0.2612	0.1867	0.0899	0.2115	***0.0768***	0.2213
Gap						–	0.2625	0.1227	0.1315	0.2681	0.1417	0.2567	0.1432
Saou							–	0.2274	0.1863	0.0369	0.1852	***0.0371***	0.1767
Ardene								–	0.0978	0.2573	0.0727	0.2365	0.1143
Luberon crete									–	0.1967	0.0492	0.1737	0.0812
Grand Luberon										–	0.2086	***0.0311***	0.2039
F.D. Lure											–	0.1909	0.0741
Sisteron												–	0.1864
F.D. Venasque													–

The variation in pairwise population differentiation with spatial distance revealed no pattern of isolation by distance. The overall increase in differentiation with distance was not significant, as shown by the regression slopes of *F*_ST_ / 1 − *F*_ST_ against the logarithm of distance b_log_*F*_ST_ = 0.028 (95% CI: −0.043 to 0.050) or *R*_ST_ / 1 − *R*_ST_ against the logarithm of distance b_log_*R*_ST_ = 0.080 (95% CI: −0.072 to 0.092). The confidence intervals are based on a permutation procedure, which randomizes the spatial locations of individuals. The test using randomization of allele sizes was not significant, suggesting that stepwise mutations did not make an important contribution to genetic differentiation (*P* = 0.1106, 95% CI: 0.0783 to 0.2965), and therefore, R_ST_ was not a better measure of differentiation than *F*_ST_ in this study.

The STRUCTURE analysis was run with *K* ranging between 1 and 10, both with and without sampling plot location to assist clustering and with and without admixture. The *K* value with the highest posterior probability was consistently *K* = 1, indicating that STRUCTURE did not detect genetic differentiation between the study sites.

### Survival analyses

Survival analyses were used to test among three scenarios generated using regional wind data (Table 3; Table S5). All of the investigated scenarios had significant and positive values of the coefficient (Table 3, *P*-value <0.0001 for all scenarios), meaning that the colonization of a site by a wasp occurred more rapidly when the covariate (here the particle point count in the grid cell of each study site according to each scenario) increased. The scenario involving long-distance dispersal from the introduction site (“Ventoux” in Table 3 and Table S5) performed better than either the scenario involving short-distance dispersal from all sites (“All Sites” in Table 3 and Table S5) or the scenario involving short-distance dispersal from all sites after a 4-year delay (“All sites plus 4-year delay” in Table 3), as indicated by the log-likelihood ratios of 54.44, 50.62, and 13.5, respectively.

**Table 3:** Results of the time-dependent survival analysis of the three windborne dispersal scenarios for *Megastigmus schimitscheki*. Positive values of the estimated effect coefficients mean that the colonization of a site occurs more rapidly when the covariate (here the particle point count in the grid cell of each study site according to each scenario) increases. The likelihood ratio test provides a *P*-value that states whether the covariate has a significant effect on the rate at which a site is colonized. Comparing likelihood ratio test values allows comparison of dispersal scenarios (because all three scenarios included the same number of parameters): the high value for the Long-distance from Mont Ventoux scenario indicates it provides the best fit to the empirical colonization data. See text for a description of the three scenarios and Table S5 for technical details of the survival analysis.

Dispersal scenarios	Estimated effect (95% confidence interval)	Likelihood ratio test	*P*-value
Long-distance from Mont Ventoux	0.0053 (0.0034, 0.0078)	54.44	<0.0001
Short-distance from all sites	0.0005 (0.0003, 0.0007)	50.62	<0.0001
Short-distance from all sites plus 4-year delay	0.0016 (0.0010, 0.0022)	13.5	0.0002

### ABC analyses of genetic data

In the DIYABC analysis, the most highly supported scenario was *DIYABC Scenario 1, Long-distance dispersal*, with a high posterior probability of 0.9729, and all other scenarios received much weaker support with non-overlapping confidence intervals (Table 4). Based on the logistic estimate, the type I error estimate for scenario 1 was 0.11, and the mean type II error estimate was 0.062 (varying from 0.031 to 0.131 depending on the true scenario) (Table 5). If scenario 1 had been frequently selected when it was not the true scenario for simulated data (high type II error rate), we could have little confidence in its selection for the observed data. However, the type II error rate was consistently lower than expected at random (0.25), which provides greater confidence that when this scenario is chosen it is likely to be the truest of the proposed scenarios. Scenarios 2, 3, and 4 all showed higher probabilities of type I error than did scenario 1, showing that they have lower power of detection. In addition, the PCA of scenario 1 showed a good fit between the posterior distribution and the real data set (Figure S2).

**Table 4:** Posterior probability of each DIYABC scenario based on the logistic estimate. Logistic regression was performed on the 40,000 simulations closest to the real value.

Scenario	Posterior probability	95% confidence interval
1	0.9729	0.9632, 0.9825
2	0.0177	0.0095, 0.0260
3	0.0042	0.0021, 0.0063
4	0.0052	0.0025, 0.0078

**Table 5:** Type I and Type II error rates when each of the DIYABC scenarios was used to simulate data and the most likely scenario was selected based on the simulated data.

Scenario	Type II error probability				Type I error probability

	1	2	3	4	
True scenario used for simulation					
1	–	0.180	0.254	0.192	0.11
2	0.031	–	0.335	0.327	0.74
3	0.024	0.352	–	0.194	0.81
4	0.131	0.194	0.302	–	0.68

Analysis of the wind direction data from all 12 local weather stations (CLIMATIK data) showed that in most years the greatest proportion of wind travelled toward the southeast. Moreover, over the seventeen year period from 1994 to 2010, the number of days during which wind travelled to the southeast was twice to three times as many as the number of days in which wind travelled in any one of the other directions (Table S9). This result coupled with the observation that the majority of the infested stands are located to the east or southeast of Mont Ventoux (Fig. 1), suggests that wind may be an important dispersal vector for this species.

## Discussion

Because the processes associated with spreading invasions are likely to be similar to those found during natural range expansions (Handley et al. 2011), the study of invasive species provides a valuable opportunity to investigate the processes associated with dispersal, including population genetic change, that can be more challenging to study in long-established native species (Rice and Sax 2005). Here, we provide a case study of a specialist invasive species that successfully expanded its range despite the highly heterogeneous and fragmented distribution of its obligatory host at the regional scale.

### The introduction area is the main source of colonizers

The central finding of our study is that using empirical spatiotemporal and genetic data for the French *M. schimitscheki* populations in three distinct analyses, we found consistent support for a long-distance dispersal pattern of invasion.

In the analysis of genetic structure, if short-distance “stepping-stone” dispersal was the main mechanism of range expansion, we would expect progressive erosion of genetic diversity and an isolation-by-distance-like pattern of differentiation. In contrast, if the main mechanism of range expansion for *M. schimitscheki* was frequent long-distance dispersal, including both dispersal between established sites and colonization of new sites, we would expect some genetic differentiation among populations due to founder effects, possibly some significant *F*_ST_ values, but little or no spatial genetic structure (Austerlitz and Garnier-Géré 2003; Bialozyt et al. 2006). The spatial genetic structure analysis found no significant isolation by distance. However, significant and non-negligible *F*_ST_ values among populations (*F*_ST_ = 0.17 on average and up to 0.34) suggest that founder effects or genetic drift may be important, and gene flow is limited. Moreover, the AMOVA analyses showed that the genetic structure is at the population level and no substructure exists at higher levels. Together, the results from the analysis of genetic structure could support range expansion through long-distance dispersal. It should also be noted that populations that are growing in effective population size may show low genetic drift and relatively stable allele frequencies even with genetic isolation, at least in the short term and depending on the genetic profile of the propagule pool (Excoffier et al. 2009). Overall, these population genetic data do not show conclusively whether or not there is frequent long-distance dispersal, but if there were continuous gene flow over short and long-distances, we would be unlikely to see significant pairwise *F*_ST_, so we suggest that substantial long-distance dispersal events occurred during population foundation.

Both the DIYABC and the survival analyses provided strong statistical support for a long-distance dispersal scenario involving the introduction area (Mont Ventoux) as the main source of all emigrants (scenarios “Ventoux” and “All from Ventoux” in Table 3 and Fig. 3, respectively). The DIYABC analysis was based on analysis of genetic data, which would have contained information on whether there has been continuous gene flow between study sites or whether sites were founded by a single dispersal event and have since been genetically isolated; however, the DIYABC model does not explicitly account for gene flow between diverged populations. Thus, if there were continuous gene flow between study sites, and if the gene flow was more frequent between neighboring populations, the DIYABC analysis would be likely to favor the short-distance dispersal scenarios over the long-distance dispersal scenarios. Thus, the statistical support for *Scenario 1* in the DIYABC analysis provides strong evidence for long-distance dispersal during *M. schimitscheki* ‘s invasion.

### A windborne invasion pattern

In insects, long-range movements may occur frequently and can lead to faster rates of spread than simple diffusion models predict (Kot et al. 1996; Suarez et al. 2001). In addition, long-distance dispersal is an essential driver of colonization success when distance to new potentially suitable sites exceeds the normal dispersal distances of species (Wilson et al. 2009a). This may especially apply to specialist species for whom both landscape fragmentation and inability to use alternative habitats represent natural barriers to simple diffusion. In our study system, the patchy distribution of the wasp's obligatory host tree suggests that the spatial scale of dispersal events must be over tens of kilometers. As the *Megastigmus* genus has not been shown to actively travel over such distances (Jarry et al. 1997), dispersal is likely to be passive. Similarly sized and closely related fig wasps have been shown to travel distances of 6–14 km (Nason et al. 1996), 40–60 km (Lin et al. 2008a,b), 100 km (Ahmed et al. 2009), and over almost 3000 km (Gardner and Early 1996).

Passive long-range movement of organisms may occur through anthropogenic or natural mechanisms, or a combination of both (Liebhold and Tobin 2008; Kerdelhué et al. 2014). Anthropogenic dispersal usually occurs from and to regions where important human activity coincides with a particular life stage that favors dispersal (e.g., Robinet et al. 2009; Kaňuch et al. 2013). In seed-specialized wasps, the life stage, which is most likely to be transported as a result of human activity, is the diapausing larval stage. Diapausing larvae remain within seeds from their harvest to their use in tree nurseries and may be transported as a result of seed trade at international, national and regional scales (Auger-Rozenberg and Roques 2012). Southeastern French cedar seeds have been attractive for seed suppliers for more than a decade, which has increased seed trade substantially throughout the French Mediterranean basin and in northern regions for both forestry and ornamental specimens (French Ministry of Food 2010). However, if seed trade was the main mechanism of long-range movement of *M. schimitscheki*, one would expect a multidirectional expansion pattern of the wasp across the French Mediterranean basin. Instead, the wasp has followed a mainly southeasterly expansion pattern away from its introduction area (Fig. 1). Asymmetry in dispersal generally occurs through a combination of environmental factors and the dispersal behavior of species (Diffendorfer 1998). Passive dispersers are influenced by directional dispersing agents such as ocean currents, river currents or prevailing winds, which usually generate a predominant flow direction biasing dispersal through a preferential direction of migration (Pringle et al. 2011). The directional pattern of *M. schimitscheki*'s expansion provides support for our proposal that wind is the most probable vector for long-distance dispersal of this species*,* as for other species (Anderson and Sturtevant 2011; Kerdelhué et al. 2014). Moreover, the regions to the east and southeast of Mont Ventoux are more mountainous and at higher elevation than the regions to the west and southwest. As wind speed is typically greater at high elevation than at low elevation (Westbrook and Isard 1999), the high elevation land to the south-east of Mont Ventoux could explain the tendency for long-distance dispersal in this direction (Bullock et al. 2002).

### Local-scale or regional-scale wind movement?

The survival analyses of the windborne expansion patterns derived from the regional-scale HYSPLIT particle dispersal model indicated that there was an informative correlation between the *M. schimitscheki* presence/absence data from the annual surveys and the scenarios based on regional-scale wind data. Indeed, although all of the dispersal models based on regional-scale wind data that were tested in the survival analysis had positive coefficient values (Table 3), the scenario involving long-distance dispersal from the introduction site (“Ventoux” in Table 3) provided more explanatory value than did the short-distance dispersal scenarios. Thus, we can say from this analysis that it is possible to construct a scenario based on regional-scale wind data that correlates with field survey data, but not that wind is necessarily the vector for dispersal.

None of the three DIYABC scenarios that explicitly included either local, near-ground wind data or regional, high-altitude data were supported in our analyses. In this case, we were unable to construct scenarios based on either wind dataset that provided more explanatory power than our simplest scenario of long-distance dispersal. This result suggests that there is a need to investigate whether other types of wind data (e.g., maximum wind speed) may be more meaningful for the construction of dispersal models.

Overall these results suggest that (1) *M. schimitscheki* appears to regularly disperse over distances greater than 10 km. Given that this distance is greater than the known active flight capacity of the genus, this dispersal is likely to be passive. (2) Our analyses of two wind datasets did not produce supported scenarios in the ABC analysis, but given the environmental importance of wind in this region, and the southeastern-tending pattern of *M. schimitscheki* dispersal, wind is still likely to be the dispersal vector. Developing methods to analyze wind as a dispersal vector remains a rich opportunity for research, for example, Nathan et al. 2011. (3) Three very different analyses, that is, traditional population genetic analysis, ABC analysis of genetic data with very simple ecological priors, and survival analysis of field survey data, produced highly congruent results. Not only does this provide confidence in the results, it offers support for our untraditional application of survival analysis and our somewhat unusual combination of independent and complementary analyses. Finally, we suggest that there is a distinct need for new analysis methods that have the capacity to combine empirical spatiotemporal field data, genetic data and environmental data to investigate dispersal and invasion.

### Implications for invasion monitoring and landscape management

Both the survival analysis and the DIYABC analysis found that long-distance dispersal based on regional-scale, high-altitude wind activity best explained the annual presence/absence data from the study sites. This result suggests that initial invasion across the range may be the result of long-distance dispersal and supports previous research which found such patterns in closely related wasps (e.g., Jarry et al. 1997; Roques et al. 2008) and other insect species (Reynolds and Reynolds 2009; Sanders et al. 2011). These results provide some guidance for the development of predictive models of invasive insect range expansion to aid in pre-emptive monitoring and control measures (Hastings et al. 2005). Given the probability of long-distance dispersal from the longest-established sites, monitoring and control efforts may be most effective if focused at the core of the spreading population rather than at the periphery of the range. However, because long-distance dispersal events have a greater impact on rates of spread than short-distance dispersal events, monitoring outlying populations remains critically important (Liebhold and Tobin 2008). In addition, because species’ range expansion is the result of combined local population growth and dispersal (Liebhold and Tobin 2008), the development of tools to predict within-site population growth would provide valuable insights into why and when a newly invaded site becomes a source of emigrants (Stanaway et al. 2011), Current work modeling *M. schimitscheki* population dynamics in relation to within-site biotic factors, such as host plant seed masting cycles and interspecific competition with the conspecific *M. pinsapinis*, will help refine our ability to focus monitoring and control efforts for this invasive wasp.

The population genetic analyses found little evidence of genetic structure, supporting the single introduction proposed by Auger-Rozenberg et al. (2012). However, globalized trade has increased accidental introductions of exotic species, and there is a particular risk in Europe from insects that develop within seeds because European Union directives have relaxed import controls on cones and seeds (EU directives 1999/105/EC and 2010/680/EU). Current seed screening methods are prohibitively labor-intensive and expensive; therefore, developing fast and inexpensive methods to identify infected seed and stop its import and trade would be an important goal for monitoring and control programs for *M. schimitscheki* and other seed and cone predators.

We suggest that current field survey methods detect populations only once they are relatively large and already serving as sources of emigrants. Monitoring programs aimed at detecting populations at an early stage of establishment would therefore need to use alternative survey techniques such as a suction trap network. Such a network could also help elucidate the effects of meteorological conditions on insect dispersal (e.g., Sanders et al. 2011) and invasion success (Hulme 2006). Moreover, as the host tree in this case is a popular ornamental, specific monitoring of ornamental specimens could provide valuable insights into the contribution of spatially isolated host individuals as stepping stones in population spread.

## Supporting Information

**Table S1.** The sites that have been sampled for *M. schimitscheki* between 1995 and 2010. **Table S2.** The primers used to genotype *M. schimitscheki*. **Table S3.** CLIMATIK climatological data stations used in this study. **Table S4.** The number of HYSPLIT models run for each study site. **Table S5.** Survival analyses to investigate how the number of particle trajectories that hit a given study site affects the time until detection of a *M. schimitscheki* population. **Table S6.** Analysis of the most probable source site for emigration to each of the study sites based on the CLIMATIK data. **Table S7.** Prior distributions for effective population size (Ne) and divergence time (t) used in the DIYABC analysis. **Table S8.** Hierarchical analysis of molecular variation at 9 microsatellite loci for 661 individuals in 14 plots. **Table S9.** The proportion of wind (km) in each of the cardinal and subcardinal directions based on all available data for the month of May between 1994 and 2010 at the 12 CLIMATIK weather stations. **Figure S1.** The National Atmospheric and Oceanographic Association HYSPLIT model predicts particle movement from a release point at a given date and time and allows particles to travel for a specified amount of time. **Figure S2.** Principal component analysis (PCA) in the space of the summary statistics performed on the selected colonization scenario (scenario 1) of *M. schimitscheki* in southeastern France.
