# Supplementary material for: Reconstruction of a windborne insect invasion using a particle dispersal model, historical wind data, and Bayesian analysis of genetic data
Source: Ecol Evol. 2014 Dec 2;4(24):4609–25. doi: 10.1002/ece3.1206 (PMC4278814; doi:10.1002/ece3.1206)
Supplement: Supplementary file 1 [file ece30004-4609-sd1.doc]

**Supporting Information**

**Supplementary Tables**

**Table S1. The sites that have been sampled for *M. schimitscheki* between 1995 and 2010.** 1 = Stand sampled and no *M. schimitscheki* individuals were found; 2 = Stand sampled and *M. schimitscheki* was found; -- = Stand not sampled, *= Stands where sampling occurred both before and after the arrival of *M. schimitscheki*.

| **Cedrus stand name** |  |  |  |  |  |  |  |  |  |  |  |  |  |  |  |  |  |  |  |  |
| --- | --- | --- | --- | --- | --- | --- | --- | --- | --- | --- | --- | --- | --- | --- | --- | --- | --- | --- | --- | --- |
|  | **Lat.** | **Long.** | **Year detected** | **4yr delay** | **1995** | **1996** | **1997** | **1998** | **1999** | **2000** | **2001** | **2002** | **2003** | **2004** | **2005** | **2006** | **2007** | **2008** | **2009** | **2010** |
| Mont Ventoux (sud) - introduction area | 44.13 | 5.26 | 1990-94 | 1995 | 2 | 2 | 2 | -- | -- | 2 | 2 | 2 | 2 | 2 | 2 | 2 | 2 | -- | 2 | 2 |
| Ventoux Jas du Mourre | 44.14 | 5.27 | 1999 | 2003 | -- | -- | -- | -- | 2 | -- | -- | -- | -- | -- | -- | -- | -- | -- | -- | -- |
| Ventoux Ramayette | 44.17 | 5.20 | 1999 | 2003 | -- | -- | -- | -- | 2 | -- | -- | -- | -- | -- | -- | -- | -- | -- | -- | -- |
| F.D. Venasque | 43.98 | 5.21 | 2000 | 2004 | -- | -- | -- | -- | -- | 2 | 2 | 2 | 2 | 2 | 2 | 2 | 2 | 2 | 2 | 2 |
| F.D. Ventouret 7 | 44.14 | 5.39 | 2000 | 2004 | -- | -- | -- | -- | -- | 2 | 2 | 2 | 2 | 2 | 2 | 2 | 2 | 2 | 2 | -- |
| Oppede | 43.83 | 5.16 | 2000 | 2004 | -- | -- | -- | -- | -- | 2 | 2 | 2 | 2 | 2 | 2 | 2 | -- | -- | -- | -- |
| Saint Lambert 1 | 44.00 | 5.29 | 2000 | 2004 | -- | -- | -- | -- | -- | 2 | 2 | 2 | 2 | 2 | 2 | 2 | 2 | 2 | 2 | -- |
| Sault | 44.11 | 5.43 | 2000 | 2004 | -- | -- | -- | -- | -- | 2 | 2 | -- | -- | -- | -- | -- | -- | -- | -- | -- |
| Barjac***** | 44.32 | 4.36 | 2001 | 2005 | -- | 1 | 1 | -- | -- | -- | 2 | 2 | 2 | 2 | 2 | 2 | 2 | 2 | 2 | 2 |
| F.D. Lure | 44.07 | 5.79 | 2001 | 2005 | -- | -- | -- | -- | -- | -- | 2 | 2 | 2 | 2 | 2 | 2 | 2 | 2 | 2 | 2 |
| Grand Luberon | 43.81 | 5.47 | 2001 | 2005 | -- | -- | -- | -- | -- | -- | 2 | 2 | 2 | 2 | 2 | 2 | -- | -- | -- | -- |
| Jabron | 44.20 | 5.76 | 2001 | 2005 | -- | -- | -- | -- | -- | -- | 2 | -- | -- | -- | -- | -- | -- | -- | -- | -- |
| Luberon crete***** | 43.80 | 5.24 | 2001 | 2005 | -- | 1 | 1 | 1 | -- | 1 | 2 | 2 | 2 | 2 | 2 | 2 | 2 | 2 | 2 | 2 |
| Menerbes***** | 43.82 | 5.22 | 2001 | 2005 | -- | -- | -- | -- | -- | 1 | 2 | 2 | 2 | 2 | 2 | 2 | -- | -- | -- | -- |
| Revest du Bion | 44.08 | 5.54 | 2001 | 2005 | -- | -- | -- | -- | -- | -- | 2 | -- | -- | -- | -- | -- | -- | -- | -- | -- |
| Sisteron | 44.24 | 5.92 | 2001 | 2005 | -- | -- | -- | -- | -- | -- | 2 | 2 | 2 | 2 | 2 | 2 | 2 | 2 | 2 | 2 |
| Ardene | 43.89 | 5.73 | 2002 | 2006 | -- | -- | -- | -- | -- | -- | -- | 2 | -- | -- | -- | -- | 2 | 2 | 2 | 2 |
| Luberon Trou du Rat***** | 43.79 | 5.13 | 2002 | 2006 | -- | -- | -- | -- | -- | -- | 1 | 2 | 2 | 2 | -- | -- | -- | -- | -- | -- |
| Saint Pierre Argencon | 44.53 | 5.69 | 2002 | 2006 | -- | -- | -- | -- | -- | -- | -- | 2 | -- | -- | -- | -- | -- | -- | -- | -- |
| Saou | 44.65 | 5.13 | 2003 | 2007 | -- | -- | -- | -- | -- | -- | -- | -- | 2 | 2 | 2 | 2 | 2 | 2 | 2 | 2 |
| Gap | 44.55 | 6.00 | 2004 | 2008 | -- | -- | -- | -- | -- | -- | -- | -- | -- | 2 | 2 | 2 | 2 | 2 | 2 | 2 |
| Foret de Barres | 44.66 | 4.73 | 2005 | 2009 | -- | -- | -- | -- | -- | -- | -- | -- | -- | -- | 2 | 2 | -- | -- | -- | -- |
| Castellane***** | 43.86 | 6.52 | 2006 | 2010 | -- | -- | -- | -- | -- | -- | -- | -- | -- | -- | 1 | 2 | 2 | 2 | 1 | 2 |
| Mirabel | 44.61 | 4.51 | 2006 | 2010 | -- | -- | -- | -- | -- | -- | -- | -- | -- | -- | -- | 2 | 2 | 2 | -- | 2 |
| Saint Ginieys en Coiron | 44.63 | 4.53 | 2006 | 2010 | -- | -- | -- | -- | -- | -- | -- | -- | -- | -- | -- | 2 | -- | -- | -- | -- |
| Saumon | 44.10 | 6.22 | 2009 | 2013 | -- | -- | -- | -- | -- | -- | -- | -- | -- | -- | -- | -- | -- | -- | 2 | 2 |
| Bauduen | 43.73 | 6.20 | 2010 | 2014 | -- | -- | -- | -- | -- | -- | -- | -- | -- | -- | -- | -- | -- | -- | -- | 2 |
| Vesc | 44.53 | 5.11 | 2010 | 2014 | -- | -- | -- | -- | -- | -- | -- | -- | -- | -- | -- | -- | -- | -- | -- | 2 |
| Alzon | 43.95 | 3.45 |  |  | -- | -- | -- | 1 | -- | -- | -- | -- | 1 | 1 | 1 | 1 | -- | -- | -- | -- |
| Breil sur Roya | 43.93 | 7.50 |  |  | -- | -- | -- | -- | -- | -- | -- | -- | -- | -- | 1 | 1 | -- | -- | 1 | -- |
| Estrechure | 44.09 | 3.80 |  |  | -- | -- | -- | 1 | -- | -- | -- | 1 | -- | 1 | -- | 1 | 1 | 1 | -- | 2 |
| F.D. Ayre | 44.43 | 3.98 |  |  | -- | -- | -- | -- | -- | -- | -- | -- | -- | 1 | -- | 1 | 1 | 1 | 1 | -- |
| F.D. Canigou | 42.59 | 2.46 |  |  | -- | -- | -- | -- | -- | -- | -- | -- | -- | 1 | -- | 1 | 1 | 1 | -- | -- |
| F.D. Espinouse | 43.58 | 2.87 |  |  | -- | -- | -- | -- | -- | -- | -- | -- | -- | 1 | -- | 1 | 1 | 1 | -- | -- |
| F.D. Fontmort | 44.25 | 3.75 |  |  | -- | -- | -- | -- | -- | -- | -- | -- | -- | 1 | -- | 1 | 1 | 1 | -- | -- |
| F.D. la Fage | 43.99 | 3.80 |  |  | -- | -- | -- | -- | -- | -- | -- | -- | -- | 1 | -- | 1 | 1 | 1 | 1 | -- |
| F.D. Lespinassiere | 43.40 | 2.53 |  |  | -- | -- | -- | -- | -- | -- | -- | -- | -- | 1 | -- | 1 | 1 | 1 | -- | -- |
| F.D. Mende | 44.49 | 3.54 |  |  | -- | -- | -- | -- | -- | -- | -- | -- | -- | 1 | -- | 1 | 1 | 1 | 1 | 1 |
| F.D. Rialsesse | 42.93 | 2.40 |  |  | -- | -- | 1 | 1 | -- | -- | -- | -- | -- | 1 | 1 | 1 | 1 | 1 | 1 | -- |
| F.D. Vis | 43.91 | 3.49 |  |  | -- | -- | -- | -- | -- | -- | -- | -- | -- | 1 | -- | 1 | 1 | 1 | -- | 1 |
| Flassan sur Issol | 43.37 | 6.22 |  |  |  |  |  |  |  |  |  |  |  |  |  |  |  |  | -- | 1 |
| G.F. Cassagnoles | 43.37 | 2.64 |  |  | -- | -- | -- | -- | -- | -- | -- | -- | -- | 1 | -- | 1 | 1 | 1 | -- | 1 |
| G.F. Pardailhan | 43.46 | 2.85 |  |  | -- | -- | -- | -- | -- | -- | -- | -- | -- | 1 | -- | 1 | 1 | 1 | 1 | 1 |
| La Garde Freinet | 43.31 | 6.47 |  |  | -- | -- | -- | -- | -- | -- | -- | -- | -- | 1 | 1 | 1 | -- | -- | -- | -- |
| Lamalou les Bains | 43.61 | 3.05 |  |  | -- | -- | -- | -- | -- | -- | -- | 1 | 1 | -- | 1 | 1 | 1 | 1 | 1 | 1 |
| Le Treps | 43.26 | 6.37 |  |  |  |  |  |  |  |  |  |  |  |  |  |  |  |  | -- | 1 |
| Marcelly | 43.36 | 2.48 |  |  | -- | -- | 1 | 1 | -- | -- | -- | -- | -- | -- | 1 | 1 | 1 | 1 | 1 | -- |
| Pelenc | 43.64 | 6.09 |  |  |  |  |  |  |  |  |  |  |  |  |  |  |  |  | -- | 1 |
| Saint André les Alpes | 44.01 | 6.47 |  |  | 1 | 1 | 1 | -- | -- | -- | -- | 1 | 1 | 1 | 1 | 1 | 1 | 1 | 1 | -- |
| Saint Maximin | 43.45 | 5.89 |  |  | -- | -- | -- | 1 | -- | -- | -- | 1 | -- | -- | 1 | 1 | -- | -- | -- | -- |
| Valliguieres | 44.02 | 4.62 |  |  | -- | -- | -- | 1 | -- | -- | -- | 1 | -- | 1 | 1 | 1 | 1 | 1 | -- | -- |
| Verignon | 43.65 | 6.27 |  |  | -- | -- | -- | -- | -- | -- | -- | -- | -- | -- | 1 | 1 | -- | -- | -- | -- |
| Vesubie | 44.08 | 7.27 |  |  | -- | -- | -- | -- | -- | -- | -- | -- | -- | -- | -- | 1 | -- | -- | -- | -- |

**Table S2. The primers used to genotype *M. schimitscheki***. The first multiplex used primers MS1-alpha, MS2-162, MW-34, MS1-110, MS3-98 and MS3-105; and the second used MS3-99, MS1-43 and MS3-91.

| **Locus** | **Primer sequence (5’-3’)** | **Forward primer dye** | **Repeat** | **Size range (bp)** | **Genbank accession no** |
| --- | --- | --- | --- | --- | --- |
| MS1-alpha | F : GACTGCAAGCTCGACTCACAC  R : TTTCTCCTCGACGCTGAT | Fam | (CA)18 | 93 – 99 | AY249163.1 |
| MS2-162 | F : GCAGACCGGCGAATAAATAA  R :TCTGCAGCACGATGTAAACG | Fam | (AG)5TG(AG)35 | 122 – 130 | AY249164.1 |
| MW-34 | F : CCCCGCCTCTACCAAATC  R : TTGAAATTGCTCGGACCG | Tamra | (AG)17(G)10 | 144 – 157 | AJ001068.1 |
| MS1-110 | F : TCAGCCCGACTTCGTCCTT  R : AGTCGGCGTTATCGGTTATT | Hex | (AG)51 | 208 – 218 | JX183085 |
| MS3-98 | F:GAGAGAGTCGCGTACGTGTAG  R :TAACGGGTGCTCGAATCAAC | Fam | (GA)28 | 203 – 213 | JX183087 |
| MS3-105 | F : ATGGTCGAGCCCGCTAC  R:GAGGGAGAGACAGACGGCAAAT | Fam | (TC)17(ACTC)5 | 260 – 272 | JX183089 |
| MS3-99 | F : ATTGACTTTGCTCTCCGTCTC  R : CCAAGTTAGCGCTTCAC | Tamra | (CT)52 | 99 – 121 | JX183088 |
| MS1-43 | F : GCAAGCCCTTCGCACAAC  R:GCGCTTCACCGACCTCC | Hex | (AC)15 | 122 – 135 | AY249162.1 |
| MS3-91 | F:CGACACTTATACACCGGCATT  R:CACGTTGCGGACAGAGAGCGA | Tamra | (TC)41 | 211 – 221 | JX183086 |

**Table S3. CLIMATIK climatological data stations used in this study.** Data were not available for all years at all stations and for the Carpentras and Embrun stations only the direction of the maximum wind event each day was available.

| Climate station | Latitude | Longitude | First year available | Last year used |
| --- | --- | --- | --- | --- |
| Alenya | 42.62 | 2.98 | 1994 | 2010 |
| Bellegard | 43.75 | 4.45 | 1996 | 2010 |
| Carpentras | 44.08 | 5.06 | 2001 | 2010 |
| Embrun | 44.57 | 6.51 | 2001 | 2010 |
| Gruissan | 43.10 | 3.09 | 1996 | 2009 |
| Marseillan | 43.35 | 3.54 | 2000 | 2010 |
| Mauguio | 43.58 | 3.96 | 2001 | 2010 |
| Montelmar | 44.59 | 4.73 | 2001 | 2010 |
| Roujan | 43.49 | 3.32 | 1994 | 2010 |
| Salon | 43.64 | 5.01 | 2000 | 2010 |
| St Marcel | 44.97 | 4.96 | 1994 | 2010 |
| Orange | 44.13 | 4.79 | 2010 | 2010 |

**Table S4. The number of HYSPLIT models run for each study site.** Using the National Oceanographic and Atmospheric Association’s HYSPLIT model trajectories were modelled for departure from each of the study sites for May 1^st^ through 31^st^ every year beginning with the year colonization was detected at each site. Sites in parentheses used data modelled for the nearest-neighbour site.

| **Site** | **year *M. schimitscheki* was detected** | **HYSPLIT model years** | **number of HYSPLIT model years** | **total days modelled** |
| --- | --- | --- | --- | --- |
| Ardene | 2002 | 2002-2010 | 9 | 279 |
| Barjac | 2001 | 2001-2010 | 10 | 310 |
| Bauduen | 2010 | 2010 | 1 | 31 |
| Castellane | 2006 | 2006-2010 | 5 | 155 |
| F.D. Lure | 2001 | 2001-2010 | 10 | 310 |
| F.D. Venasque | 2000 | 2000-2010 | 11 | 341 |
| F.D. Ventouret 7 | 2000 | 2000-2010 | 11 | 341 |
| Foret de Barres (Menerbes, Saint Ginieys en Coiron) | 2005 (2001, 2006) | 2005-2010 | 6 | 186 |
| Gap | 2004 | 2004-2010 | 7 | 217 |
| Grand Luberon (Luberon Trou du Rat**)** | 2001 (2002) | 2001-2010 | 10 | 310 |
| Jabron | 2001 | 2001-2010 | 10 | 310 |
| Luberon crete | 2001 | 2001-2010 | 10 | 310 |
| Mirabel | 2006 | 2006-2010 | 5 | 155 |
| Mont Ventoux (Ventoux Jas du Mourre, Ventoux Ramayette) | 1995 (1999, 1999) | 1995-2010 | 16 | 496 |
| Oppede | 2000 | 2000-2010 | 11 | 341 |
| Revest du Bion | 2001 | 2001-2010 | 10 | 310 |
| Saint Lambert | 2000 | 2000-2010 | 11 | 341 |
| Saint Pierre Argencon | 2002 | 2002-2010 | 9 | 279 |
| Saou | 2003 | 2003-2010 | 8 | 248 |
| Sault | 2000 | 2000-2010 | 11 | 341 |
| Saumon | 2009 | 2009-2010 | 2 | 62 |
| Sisteron | 2001 | 2001-2010 | 10 | 310 |
| Vesc | 2010 | 2010 | 1 | 31 |
| **Total** |  |  | **194** | **6014** |

**Table S5: Survival analyses to investigate how the number of particle trajectories that hit a given study site affects the time until detection of a *M. schimitscheki*** **population.** We accounted for the interval censored data by considering that a sample at time *t*_2_ in a site for which the last sampling occurred at time *t*_1_ can result in finding *M. schimitscheki,* with probability $\left(1-e^{-\lambda\left(t_{1},t_{2}\right)}\right)$, or not finding *M. schimitscheki,* with probability $e^{-\lambda\left(t_{1},t_{2}\right)}$. These equations rely on the assumption that the successful establishments occur following a Poisson process. $\lambda\left(t_{1},t_{2}\right)$ was successively taken as (i) $\lambda\left(t_{1},t_{2}\right)=\alpha\times\left(t_{2}-t_{1}\right)$, null hypothesis (H0) where the rate of successful establishments is constant through time, (ii) $\lambda\left(t_{1},t_{2}\right)=\alpha\times\left(t_{2}-t_{1}\right)+\beta\sum_{t=t_{1}}^{t_{2}} NP\left(t\right)$, where *NP*(*t*) is the number of particle trajectories that hit the site considered during year *t*; this Model 1 (M1) hypothesis considers that the rate of successful establishments is proportional to the number of trajectories that hit the site considered between time t1 and t2, (iii) $\lambda\left(t_{1},t_{2}\right)=\alpha\times\left(t_{2}-t_{1}\right)+\beta\sum_{t=1994}^{t_{2}} NP\left(t\right)$; this Model 2 (M2) hypothesis considers that the detection at time t2 is proportional to the number of particle trajectories that hit the site considered since the introduction in 1994, even if the successful establishment has not been detected at time *t*_1_.

For models M1 and M2, the trajectories considered can be those originating from the initial introduction site Mont Ventoux (Ventoux), from any colonized study site (All) or from any colonized study site starting 4 years after the site was identified as colonized (All plus four-year delay) (see Material and Methods section for details).

We estimated the parameters of the different models using a maximum likelihood implemented in Mathematica. The effects of the different variables in models M1 and M2 were obtained by comparing the fits with the null model H0 through a likelihood ratio test. Likelihood Ratio Tests indicate that (i) the model M2 better fit the data, and (ii) for both models M1 and M2, the long-distance dispersal hypothesis (particle trajectories starting from Mont Ventoux better explain the data.

| Model | Number of particle trajectories | Estimated effect (α or β)  [95%-confidence interval] | Log-likelihood | Likelihood Ratio Test | P-value |
| --- | --- | --- | --- | --- | --- |
| H0 | -- | 0.057  [0.038, 0.082] | -58.98 | -- | -- |
| M1 | Ventoux | 0.0053  [0.0034, 0.0078] | -31.76 | 54.44 | <0.0001 |
|  | All Sites | 0.0005  [0.0003, 0.0007] | -33.67 | 50.62 | <0.0001 |
|  | All sites plus four-year delay | 0.0016  [0.0010, 0.0022] | -52.23 | 13.5 | 0.0002 |
| M2 | Ventoux | 0.0026  [0.0017, 0.0037] | -41.27 | 35.42 | <0.0001 |
|  | All Sites | 0.0003  [0.0002, 0.0004] | -44.20 | 29.56 | <0.0001 |
|  | All sites plus four-year delay | 0.0009  [0.0006, 0.0013] | -56.64 | 4.7 | 0.03 |

**Table S6. Analysis of the most probable source site for emigration to each of the study sites based on the CLIMATIK data.** The probability of a new *M. schimitscheki* colonization at each of the uncolonized study sites due to immigration from any one of the already colonized sites was estimated based on the presence or absence of wind from the source site towards the uncolonized site in the year prior to detecting colonization at the uncolonized site in the annual surveys. The wind data from the nearest CLIMATIK station was used for each source site.

| Year | ID | Uncolonized site | Possible source site(s) | Not possible source site(s) | CLIMATIK station used |
| --- | --- | --- | --- | --- | --- |
|  | VT | Ventoux | Introduction |  |  |
| 1999 | VQ | Venasque | VT | -- | Carpentras |
| 2000 | GL | Grand Luberon | VT,VQ, | -- | Carpentras |
| 2000 | LC | Luberon Crete | VT,VQ, | -- | Carpentras |
| 2000 | SI | Sisteron | VT,VQ, | -- | Carpentras |
| 2001 | AR | Ardene | VT,VQ,GL,LC | -- | Salon |
| 2002 | SA | Saou | VT,VQ,GL,LC | SI,AR | Carpentras, Montelimar |
| 2003 | GA | Gap | SI,AR | VT,VQ,GL,LC,SA | Carpentras, Montelimar |
| 2004 | FB | F. de Barres | SA,GA | VT,VQ,GL,LC | Montelimar, Embrun |
| 2005 | CA | Castellane | SI,GA | VT,VQ,GL,LC,AR | Carpentras, Salon |
| 2005 | MI | Mirabel | GL,LC | VT,VQ,SI,AR,SA,GA,FB,CA | Carpentras, Montelimar, Salon |
| 2008 | SM | Saumon | All | -- | Carpentras |
| 2009 | VS | Vesc | VT,VQ,GL,LC,SI,SA,GA | AR,FB,CA,MI | Carpentras, Montelimar, Embrun |
| 2009 | LU | Lure | VT,VQ, | -- | Carpentras |

**Table S7.** **Prior distributions for effective population size (Ne) and divergence time (t) used in the DIYABC analysis.** Uniform distributions were used for both demographic and time parameters, expect for N17, which Ne was drawn in a log-uniform distribution.

|  |  | Effective population size (Ne) | |
| --- | --- | --- | --- |
| Population | Year of first detection | Prior range minimum | Prior range maximum |
| N1: Mont Ventoux | 1995 | 10 | 10 000 |
| N2: Vesc | 2010 | 10 | 2 000 |
| N3: Saumon | 2009 | 10 | 2 000 |
| N4: Mirabel | 2006 | 10 | 5 000 |
| N5: Castellane | 2006 | 10 | 5 000 |
| N6: Foret de Barres | 2005 | 10 | 5 000 |
| N7: Gap | 2004 | 10 | 5 000 |
| N8: Saou | 2003 | 10 | 5 000 |
| N9: Ardene | 2002 | 10 | 5 000 |
| N10: Luberon Crete | 2001 | 10 | 10 000 |
| N11: Grand Luberon | 2001 | 10 | 10 000 |
| N12: F.D. Lure | 2001 | 10 | 10 000 |
| N13: Sisteron | 2001 | 10 | 10 000 |
| N14: F.D. Venasque | 2000 | 10 | 10 000 |
| N15: historic ‘source population’ 1 |  | 10 | 10 000 |
| N16: historic ‘source population’ 2 |  | 10 | 10 000 |
| N17: bottleneck at Mont Ventoux |  | 10 | 1 000 |
|  |  | Time step (generations) | |
| t1 (foundation of N1) |  | 12 | 15 |
| t2 (admixture between N15 and N16) |  | 12 | 1 000 |

**Table S8. Hierarchical analysis of molecular variation at 9 microsatellite loci for 661 individuals in 14 plots. Variance in allelic frequencies among plots was analysed using unordered alleles (F_ST_ like AMOVA) and alleles ordered according to their size (R_ST_ like AMOVA) in GenAlEx [26]. For each source of variation we give: the number of degree of freedom (df); the sum of squared difference to the mean (SS), variance components (VC) and the percentage of total variance (%TV). Below, the F statistics and R statistics for the 14 study sites**

|  |  | Fst like AMOVA | | | Rst like AMOVA | | |
| --- | --- | --- | --- | --- | --- | --- | --- |
| Source of variation | df | SS | VC | % TV | SS | VC | % TV |
| Among populations | 13 | 523.99 | 0.47 | 17.11 | 15206.17 | 13.09 | 10.59 |
| Among individuals | 647 | 2978.10 | 2.27 | 82.89 | 144556.60 | 110.51 | 89.41 |

|  | Observed value | Mean permuted value | 95% CI | |  | Observed value | Mean permuted value | 95% CI | |
| --- | --- | --- | --- | --- | --- | --- | --- | --- | --- |
| Fst | 0.1721 | -0.0002 | -0.0028 | 0.0033 | Rst | 0.1063 | 0.0002 | -0.0054 | 0.0078 |
| Fis | 0.0488 | 0.0002 | -0.0201 | 0.0204 | Ris | 0.1423 | -0.0010 | -0.0434 | 0.0543 |
| Fit | 0.2125 | -0.0006 | -0.0174 | 0.01789 | Rit | 0.2334 | -0.0003 | -0.0327 | 0.0334 |

**Table S9. The proportion of wind (km) in each of the cardinal and sub-cardinal directions based on all available data for the month of May between 1994 and 2010 at the 12 CLIMATIK weather stations.** The direction with the largest proportion each year is in bold and italic. The final row shows the number of days in May during the 17 year period when wind travelled in each of the directions.

| Year | N | NE | E | SE | S | SW | W | NW |
| --- | --- | --- | --- | --- | --- | --- | --- | --- |
| 1994 | 0.19 | 0.04 | 0.14 | ***0.33*** | 0.05 | 0.05 | 0.08 | 0.12 |
| 1995 | 0.23 | 0.02 | 0.20 | 0.22 | 0.00 | 0.00 | 0.06 | ***0.27*** |
| 1996 | 0.17 | 0.04 | 0.14 | ***0.41*** | 0.03 | 0.07 | 0.12 | 0.01 |
| 1997 | 0.09 | 0.11 | ***0.24*** | 0.23 | 0.05 | 0.05 | 0.13 | 0.10 |
| 1998 | 0.00 | 0.02 | 0.11 | ***0.53*** | 0.05 | 0.06 | 0.10 | 0.14 |
| 1999 | 0.01 | 0.03 | 0.13 | ***0.57*** | 0.04 | 0.04 | 0.07 | 0.11 |
| 2000 | 0.00 | 0.06 | 0.12 | ***0.33*** | 0.07 | 0.07 | 0.13 | 0.23 |
| 2001 | 0.08 | 0.10 | 0.04 | ***0.29*** | 0.12 | 0.14 | 0.09 | 0.14 |
| 2002 | 0.12 | 0.11 | 0.04 | ***0.28*** | 0.06 | 0.10 | 0.12 | 0.16 |
| 2003 | 0.12 | 0.10 | 0.05 | ***0.30*** | 0.09 | 0.10 | 0.15 | 0.10 |
| 2004 | 0.17 | 0.11 | 0.03 | ***0.27*** | 0.11 | 0.13 | 0.12 | 0.06 |
| 2005 | 0.10 | 0.08 | 0.09 | ***0.21*** | 0.09 | 0.12 | 0.16 | 0.16 |
| 2006 | 0.15 | 0.03 | 0.03 | ***0.23*** | 0.14 | 0.17 | 0.11 | 0.14 |
| 2007 | 0.11 | 0.10 | 0.06 | 0.15 | 0.13 | ***0.18*** | 0.12 | 0.15 |
| 2008 | 0.08 | 0.07 | 0.15 | ***0.28*** | 0.14 | 0.13 | 0.07 | 0.09 |
| 2009 | 0.14 | 0.14 | 0.10 | 0.14 | 0.12 | 0.10 | 0.09 | ***0.17*** |
| 2010 | 0.16 | 0.12 | 0.06 | 0.10 | 0.08 | 0.10 | 0.13 | ***0.24*** |
| Proportion, all years | 0.11 | 0.09 | 0.08 | ***0.27*** | 0.09 | 0.11 | 0.11 | 0.13 |
| Day count | 717 | 570 | 559 | 1785 | 620 | 719 | 740 | 889 |

**Supplementary Figures**


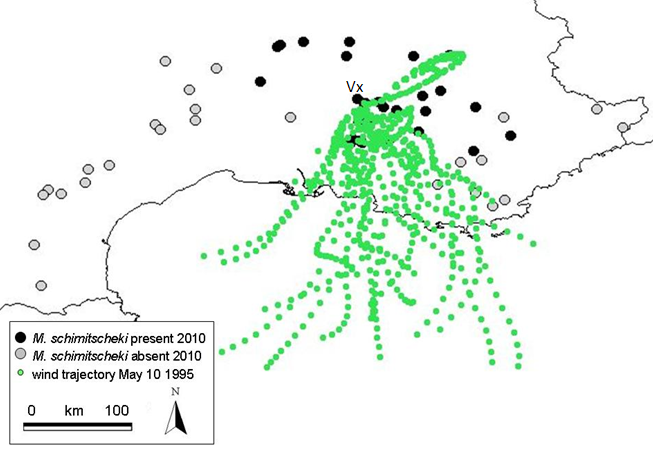


**Figure S1. The National Atmospheric and Oceanographic Association HYSPLIT model predicts particle movement from a release point at a given date and time and allows particles to travel for a specified amount of time.** The figure shows an example of predicted particle dispersal from Mont Ventoux (Vx) if the particle was released at 6am on May 10, 1995 and allowed to travel for 15 hours.


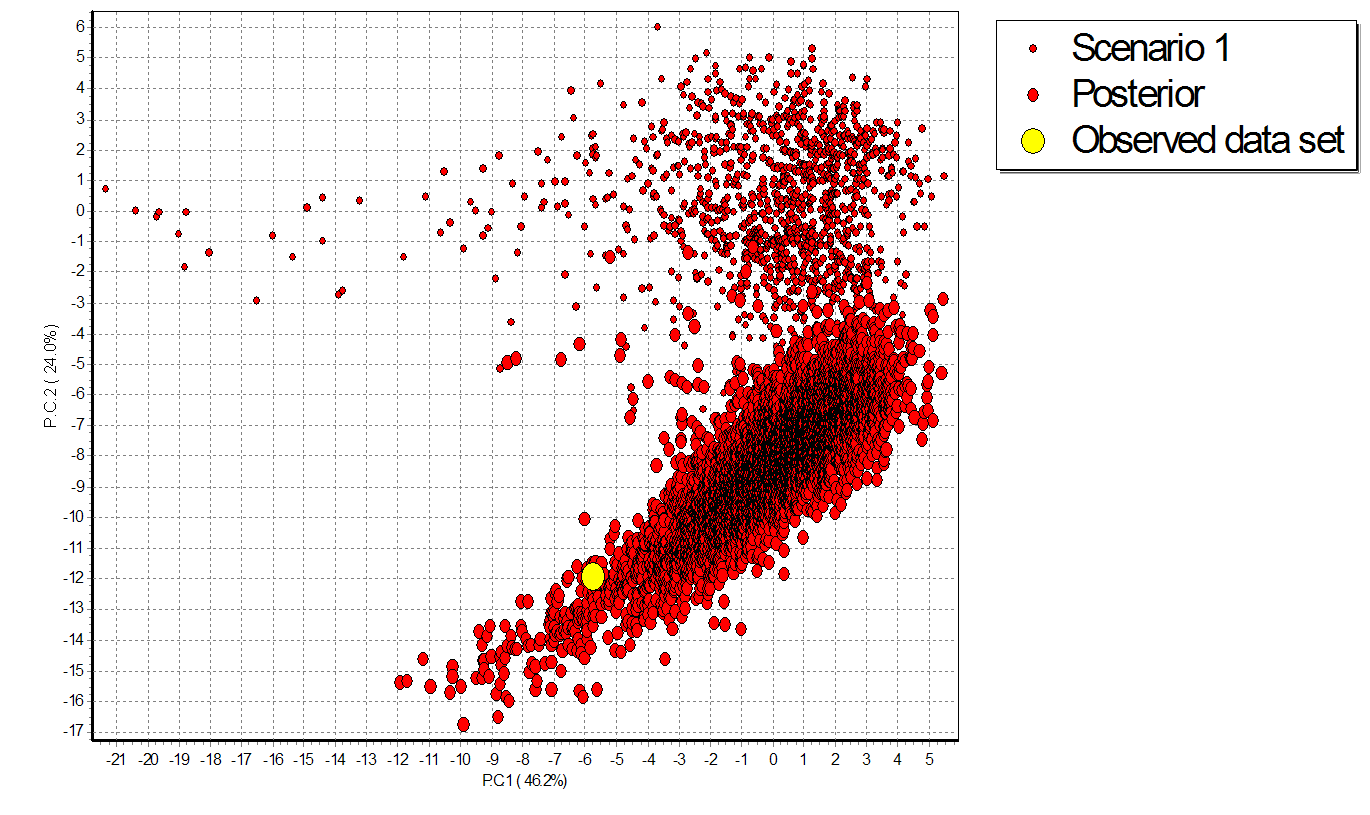


**Figure S2.** **Principal component analysis (PCA) in the space of the summary statistics performed on the selected colonization scenario (scenario 1: long distance dispersal) of *M. schimitscheki* in southeastern France.** The observations are the simulated data sets and the variables are the summary statistics. The yellow dot corresponds to the real data set of *M. schimitscheki*. Each large red dot corresponds to a dataset simulated with parameters drawn from the posterior distributions. Each small red dot corresponds to a dataset simulated with parameters drawn from the prior distributions. 5000 datasets are randomly shown here for each of the prior and posterior distributions.
